# Congenital septal defects in Karachi, Pakistan: an update of mutational screening by high-resolution melting (HRM) analysis of *MTHFR C677T*

**DOI:** 10.1186/s40246-023-00566-5

**Published:** 2024-01-29

**Authors:** Syed Irtiza Ali, Obaid Yusuf Khan, Nadir Naveed, Hussain Ahmad, Najma Patel, Afsheen Arif

**Affiliations:** 1 The Karachi Institute of Biotechnology and Genetic Engineering, University of Karachi, Karachi, Pakistan; 2 Department of Genetics, University of Karachi, Karachi, Pakistan; 3 National Institute of Cardiovascular Diseases, Karachi, Pakistan

**Keywords:** Congenital septal defects, High-resolution melting, *MTHFR* gene, Missense mutation *C677T*

## Abstract

**Background:**

Congenital heart defects (CHDs) are the heart structural malformations present at birth. Septal defects account for 40% of CHD, including atrial, ventricular and atrioventricular septal defects. In Pakistan, the prevalence of CHD is 3.4 in 1000, and a study estimated that 60,000 babies are born with CHD annually. Methylenetetrahydrofolate reductase (MTHFR), a chief enzyme, involved in the folate metabolism. The missense mutation, *C677T* (rs1801133), exists in *MTHFR* gene, results in a *MTHFR* thermolabile variant having low enzymatic activity. The study is aim to identify the *MTHFR C677T* variant association with septal defects.

**Methods:**

Samples of 194 CHD patients (age $\overline{X }\hspace{0.17em}$= 5.8 ± 5.1) and 50 normal echo controls (age $\overline{X }\hspace{0.17em}$= 6.0 ± 4.9), confirmed by pediatric consultant, were collected. Extracted DNA, quantified by agarose gel electrophoresis and nanodrop, was screened for SNP by high-resolution melting (HRM). Further, HRM results were confirmed using restriction analysis and sequencing. HRM was simply and precisely genotyped the samples within 3 h at low cost.

**Results:**

Genotypic data suggested that heterozygous mutant (CT) was frequent in congenital septal defect patients (0.26) which was higher than controls (0.143), *p* > 0.05. Mutant (TT) genotype was not found in this study.

**Conclusions:**

rs1801133 has lack of significant association with congenital septal defects. The absence of TT genotype in this study suggesting the role of natural selection in targeted population. HRM is an easy, fast and next generation of PCR, which may be used for applied genomics.

**Supplementary Information:**

The online version contains supplementary material available at 10.1186/s40246-023-00566-5.

## Introduction

Congenital heart defect or CHD affects 1% of the total live birth (about 1 million people annually) and becomes a leading birth defect [1]. It is a defect, present at birth, in the structure and regulation of heart [2]. The prevalence of CHD was surprisingly increased from 11 to 57% [3], the incident rate has also changed to 10–12 per 1000 live births and became an alarm to global health administrates [4–6]. According to a systematic review, 10% CHD prevalence increases in every 5 years [7]. More than 25 types of CHDs are reported, among them about 40% accounts for septal malformation of heart. Septal malformations are the hole in the heart septa which differentiate the heart chambers. These malformations include atrial septal defects (ASDs), ventricular septal defects (VSDs) and atrioventricular septal defects (AVSD) [6, 8]. In Karachi (largest and well-populated city of Pakistan), a tertiary care hospital has been reported that about 60,000 babies are born with these malformations, out of them 60% died in early age [9]. Therefore, it has been assumed that a wide spectrum of Pakistani population is survived with CHDs.

Methylenetetrahydrofolate reductase *MTHFR* gene (OMIM # 607,093) has been studied since the past two decades, holds eighth position in research, related 3256 or more articles were published [10, 11], due to its involvement in folate metabolism, synthesis of DNA and homocysteine [12, 13]. *MTHFR* gene loci are p(short) arm of chromosome 1 which contains 12 exons. This 21.198-kb long gene encodes a protein having 656 amino acid (74.79 kDa in weight). A missense thermolabile variant of *MTHFR* gene is *rs1801133* at C*677T*, which results in the amino acid substitution of alanine (A) to valine (V), located at 226 in the protein sequence [14]. This alteration of catalytic region of exon 4 decreases 50–70% MTHFR enzyme activity, if homozygous mutant (TT) genotype is observed [15–17]. Moreover, an influence of retardation of MTHFR enzyme activity (~ 30%) is observed if genotype is heterozygote (CT) [17, 18]. It has been found that a rise of homocysteine level causes homocysteinemia, an elevation in concentration of red cells folate and lowering the concentrations of plasma folate is strongly associated with *rs1801133* [19]. This mutation has widely studied with different diseases and has revealed a strong association with cardiovascular diseases in Asian [20, 21], including Pakistani population [22]. A number of investigations summarize the toxicity of epigenetic factors particularly folic acid in progression of congenital heart defects [18, 23, 24]. Still, this common gene has not any study on association analysis of congenital septal defects in Pakistani population.

The previous studies shown a vast variety of factors that are associated to the risk of septal defects. This study found that one of them is consumption of tobacco products practiced by at least one of the parents. Parents, who consumed a lot of these toxic products, may have greater chance of heritable gene modifications, which may depend on the passage of exposure time. In the current study, parental cigarette usage presented unique percentage among market-available tobacco products, may be a potential cause of CHD. Second-hand smoke is an another significant cause and may have influence on genetic instability when a pregnant woman is continuously exposed to passive smoking, and may create harmful environment for the child in mother’s womb. In parallel with, Naswar (addiction often found in Pashtun population) had a greater influence to cause the disease, because Pashtun was the second largest affected population in this research. In addition, Naswar was the second most common parental tobacco practice among the population of the current study; hence, it was concluded that Naswar was the common risk factor next to the smoking. Furthermore, Gutka or Mawa, wet or dry tobacco, respectively, with powder of areca nuts, paraffin wax and some sweet flavors, practicing represented a serious and rising issue than the cigarette. In particular to this, it has been found that some parents almost 6% was consuming more than one type of tobacco products. Briefly, 40% subject’s parents were addicted toward tobacco products and practiced on regular basis, which revealed an alarming situation of these toxic products (data were not provided). Additionally, more studies are required on this topic.

### HRM optimization—an update of molecular screening

High-resolution melting (HRM) genotyping is a fast, simple and an advance PCR-based mutational screening strategy without post-PCR techniques [18, 25, 26]. The new innovations in hybridized labeled probes, they produce fluorescence when bind ssDNA [27], provide more data points based on the composition, size and amount of GC content of DNA [28]. HRM analysis, since its establishment (2003), is most popular technique in western countries achieving thousand works using HRM from bacteria to human. This technique has been used in significant research of multiple disciplines; however, to date, very few studies have been conducted regarding HRM in Pakistan and become the initial study of genotyping using HRM in Pakistan. Therefore, the current study aims to find the *MTHFR C677T* association with congenital septal defect Pakistani patients using HRM. This study aims toward the establishment and optimization of *MTHFR C677T* genotyping using HRM. This was done via Bio-Rad CFX96™ system with Precision Melt Analysis™ software.

### Real-time PCR cycle optimization

Initially, PCR-HRM was performed with no cycle extension (two-step). These default parameters did not provide good and suitable amplification. Therefore, PCR-HRM was optimized with cycle extension (three-step). The reason of three-step process, which was guided by manufacturer, was GC-rich content. The amplification plots, obtained from this parameter, generated better plots than the two-step default method. Thus, three-step PCR (addition of cycle extension) was used in later experiments.

### Cooling effect optimization

This effect is crucial to cause heteroduplexes formation (between ssDNA and fluorescent dye). This effect produces dissociation curve when denatured DNA (ssDNA) accumulates the fluorescent dye. It could be achieved by heating with extreme temperature (~ 95 °C), to make ssDNA, then cool reaction down to 40 or 60 °C forming heteroduplexes. In this study, 60 °C/min was optimized in generating better melt profiles. The difference in PCR (thermal cycler) system, the dye and the targeted gene may limit this effect [18, 29].

### HRM temperature increments optimization

Tm shift has its importance in HRM genotyping. The homozygous variants are categorized on x-axis with the temperature alteration, while change in shape of melt curve is the characteristic of heterozygous variants. In this study, the optimized HRM temperature increments were 0.2 °C/10 s, heated slowly to 65–95 °C. The different SNP classes and experimental setups may alter these increments.

### MgCl_2_ concentration optimization

The activity of Taq polymerase can be achieved by optimized MgCl_2_ concentration. In the current study, MgCl_2_ gradient (0.5–3 M) was performed. Amplification was found better in 1.5 and 3 M. However, 1.5 M MgCl_2_ concentration was used in later experiments, because good amplification plot and HRM results were observed than in 3 M MgCl_2_. Still, a study stated that the use of 3 M MgCl_2_ concentration had good amplification [30].

### Sequencing

Sanger sequencing, a gold standard technique, was used for the confirmation and verification of the PCR–RFLP results. These samples were applied as positive control for HRM analysis. Additional file 1: Fig. S1 shows Sanger sequencing chromatogram of the homozygous wildtype genotype (CC). Additional file 1: Fig. S2 shows that the single-nucleotide alteration leads to the alteration of amino acid in heterozygous genotype. Homozygous mutant (TT) genotype was not found in any randomized sequences (reason is described under the heading of genotypic frequency).

## Methodology

### Sample size calculations

The sample size was designed by the given formula, where SS = sample size, *Z* = *Z*-score, *P* = prevalence of the disease (population proportion), and C.I = 95% confidence interval.

$${\text{SS}} = \frac{{Z^{2} \times P \times \left( {1 - P} \right)}}{{\left( {{\text{C}}.{\text{I}}} \right)}}$$

$${\text{SS}}_{{\left( {{\text{ASD}}} \right)}} = \frac{{3.8416 \times 0.003 \times \left( {1 - 0.003} \right)}}{{\left( {0.000625} \right)}} = 18$$

$${\text{SS}}_{{\left( {{\text{VSD}}} \right)}} = \frac{{3.8416 \times 0.004 \times \left( {1 - 0.004} \right)}}{{\left( {0.000625} \right)}} = 24$$

$${\text{SS}}_{{\left( {{\text{AVSD}}} \right)}} = \frac{{3.8416 \times 0.0005 \times \left( {1 - 0.0005} \right)}}{{\left( {0.000625} \right)}} = 3$$

### Ethical approval and sample collection

The institutional review board of The Karachi Institute of Biotechnology and Genetic Engineering (KIBGE), University of Karachi and National Institute of Cardiovascular Disease (NICVD), was given the ethical approval for conducting this case–control study. The experiments during this presented study were followed the Declaration of Helsinki. Blood samples of each septal defect (101 males and 91 females) were collected after taking informed consent and questionnaire from the patient’s guardian. Patients of age (5.8 ± 5.1) with weight (16.3 ± 11.5) and controls of age (6.0 ± 4.9) with weight (18.1 ± 12.9) having non-syndromic and non-familial case study were included, after the diagnoses through echo and consultant advice. The fifty normal echo controls were included for the sake of the small ASDs that are not usually diagnose in early ages and the complications hide till 45 years of age in some cases [31–33].

### Genomic DNA extraction

DNA was extracted using salting out method, from 1-ml blood [34]. Briefly, cell lysis was achieved by thrice the volume red cell lysis buffer (RCLB) and centrifuge for 13,000 rpm for 60 s. The pellets were washed by 1-ml deionized water and centrifuge for 13,000 rpm for 60 s. The pellets were suspended in a solution (containing 30-µl proteinase K enzyme, 100-µl 10% SDS solution, 160-µl proteinase K buffer solution and 480-µl autoclaved distilled water) and incubated at 55 °C for 2 h. Salting out was carried by NaCl (100 µl added into the solution), and the supernatant was collected after centrifugation for 5 min at 13,000 rpm. DNA was precipitated by adding absolute ethanol (99%) and inverted mix until DNA threads were observed. This solution was then centrifuged for 2 min at 13,000 rpm, and the pellets were washed with 1-ml chilled 70% ethanol. TE buffer was added in the DNA pellets to store the DNA. The quality and quantity of DNA was checked by Nanodrop analyzer (IMPLEN NanoPhotometer^®^ P-Class Germany) and agarose gel electrophoresis, respectively.

### PCR–RFLP

PCR amplification was carried by 50-ng/µL DNA template. Reported primer sequences (MTHFR-1 and 2) were used (Table 1) [18]. The optimal PCR reaction was 25 μl including: 3-μl genomic DNA, 1-μl pair of primer MTHFR-1 and 2, 12 μl of Master Mix Thermo ScientificTM, US (containing optimized dNTPs, green buffer, MgCl2 and Taq polymerase and is commercially available) and 9-μl nuclease-free water. The amplified product of size 282 bp was visualized using agarose gel electrophoresis under Gel Documentation System (FastGene^®^ FAS V, nippon genetics, Germany). Restriction analysis was achieved by Hinf I 10U/µl (Thermo Scientific™, US), following incubation for 16 h for enzyme digestion.

**Table 1:** Primer sequences and the amplicon size for PCR reaction

Primers	Primer sequences	Product size	Genotyping technique
MTHFR-1	5′-GCCTCTCCTGACTGTCATCC (Exonic)	282 bp	RFLP and sequencing
MTHFR-2	5′AGGACGGTGCGGTGAGAGTG (Intronic)		
MTHFR-3	5′**-**GAAGGAGAAGGTGTCTGCG (Exonic)	45 bp	HRM

### Sequencing

Random sample was selected based on RFLP analysis and send to MoleQule-On for performing Sanger sequencing. Sequencing was used as gold standard, and these samples were used as controls for HRM genotyping. Purification of samples was performed using columns-based purification. Data have been visualized via electropherogram software version CLC, etc.

### HRM analysis

High-resolution melt analysis was done via Bio-Rad CFX96™ system. Precision Melt Analysis™ was used to analyze melt curve. Optimization of HRM analysis was performed by using reported Primers named as MTHFR-3 and 4 [29]. The optimal real-time PCR reaction was 10 μl including: 1-μl genomic DNA, 1-μl pair of primer MTHFR-3 and 4, 6-μl Precision Melt Supermix, Bio-Rad (active ingredients were hot-start iTaq™ polymerase and EvaGreen dye along with dNTPs) and 0.6 μl of 2.5-mM MgCl_2_ with 2.4-μl volume of nuclease-free water.

### Bioinformatics analyses

Multiple sequence alignment and generation of chromatograms for mutational screening were done using Mega 7. Online accessible tool Ensemble (https://asia.ensembl.org/Homo_sapiens/ Variation/Sequence) was used to retrieve reference sequence. NCBI and Ensemble were used to observe global genotypic and allelic frequency distribution of *rs1801133* in 1000 genome project, and comparison of our data with these frequencies was performed.

### Statistical analyses

Plotting of graphs and for data storage about individual’s information Microsoft Excel was used. Calculations of mean, standard deviation and Fisher’s exact test were performed by SPSS Statistics 17.0 (Chicago, IL, USA). Odds ratio was calculated by online accessible statistical tool, MedCalc (https://www.medcalc.net/statisticaltests/odds_ratio.php).

## Results and discussion

The outcome of overall 194 individuals, containing cases of 50 each ASD and VSD, 44 AVSD and 50 controls, is presented and genotype distribution followed the Hardy–Weinberg equilibrium.

### Septal defects profiling

Congenital septal defects have been recognized as ASD, VSD and AVSD. These septal malformations account for almost 40%-50%, and this can be changed whenever time, ethnicity and geographical location, etc., are different [6, 35, 36]. In this present study, the majority of ASD population have large secundum-type ASD (OMIM#108800) (46%), whereas smaller proportion of PFO (OMIM#610338) (0.04%) and sinus venosus-type ASD (OMIM#108900) (0.06%). Furthermore, isolated primum ASD was not observed whereas complex primum ASD was quite common, showing the disease severity. Also, primum ASD (OMIM#606215) is frequently the symptom of AVSD. In addition, the VSD population have more patients with small peri-membranous VSD (OMIM#614429) (32%) while small group of patients with supracristal VSD, misaligned VSD and multiple VSD (0.04%). VSD with large peri-membranous and muscular subtype was also commonly appeared with different types of CHD, particularly with AVSD. Further, complex AVSD (38.6%) was more frequent type of AVSD; however, transitional and intermediate AVSD contributed (2.3%) a smaller proportion of data. Table 2 illustrates the distribution of septal defects (ASD, VSD and AVSD) in this study.

**Table 2:** Distribution of septal defects among different septal defect subtypes

S. No.	Septal defect	Subtype of septal defect	Number of patients	Contribution in subsets (%)	%Age in total data of CHD (*n* = 144)
01	ASD (*n* = 50)	ASD II	31	62	21.5
		ASD IV	01	2	0.69
		PFO	02	4	1.39
		Surgical close	05	10	3.47
		Complex septal defect (ASD with other CHD)	11	22	7.64
02	VSD (*n* = 50)	Peri-membranous	21	42	14.6
		Muscular	07	14	4.86
		Supracristal	02	4	1.39
		Misaligned	02	4	1.39
		Multiple	02	4	1.39
		Surgical close	06	12	4.17
		Complex septal defect (VSD with other CHD)	10	20	6.9
03	AVSD (*n* = 44)	Complete	13	29.5	9.03
		Partial	07	15.9	4.86
		Transitional	01	2.27	0.69
		Intermediate	01	2.27	0.69
		Surgical close	05	11.36	3.5
		Complex septal defect (AVSD with other CHD)	17	38.6	11.8

*ASD* Atrial septal defect, *VSD* Ventricular septal defect, *AVSD* Atrioventricular septal defect, *ASD II* Secundum ASD, *ASD IV* Coronary sinus septal defect and *PFO* Patent foreman ovale

### CHD is a multiple trait disease

#### Ethnicity

This recent study was conducted in a metropolitan city Karachi, where all Pakistani ethnicities live together because the availability of various employment opportunities. Thereby, out of 144 congenital septal defects patients, twelve different ethnicities were randomly found for the septal defects. Table 3 shows the frequencies of ethnicities, in the data, the Sindhi population found to be more affected, followed by Pashtun and native heterogenous. However, Baloch and Punjabi had similar ratio and followed by Hazara. The Sindhi population had high percentage, it may because of the study’s sampling was from Karachi (Sindh); hence, Sindhi population, around the corners, is the major population. Few studies limit the CHD within certain ethnicity; however, CHD is concluded as multifactorial trait [37, 38].

**Table 3:** Distribution of patients among different ethnicities

No.	Ethnicity	Frequency	%Age (*n* = 144)	S. No.	Ethnicity	Frequency	%Age (*n* = 144)
01	Sindhi	48	33	07	Saraiki	5	3.4
02	Pashtun	23	15.9	08	Barohi	3	2.7
03	Heterogenous	21	14.5	09	Memon	3	2.7
04	Punjabi	14	9.7	10	Balti	2	1.4
05	Baloch	13	9	11	Bengali	2	1.4
06	Hazara	9	6.2	12	Gujrati	1	0.7

### Molecular screening of *MTHFR C677T*

#### PCR–RFLP

**Fig. 1:**
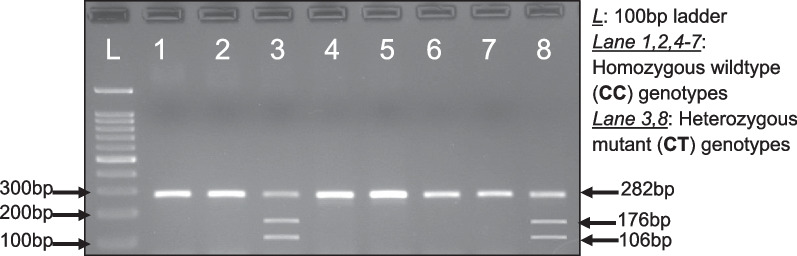
Gel image of restriction analysis by Hinf I digestion. Lanes 1, 2 and 4–7 show only one band, which confirms the sample contain homozygous genotype whereas lanes 3 and 8 have showed three bands (106, 176 and 282 bp), and it confirms that the sample has heterozygous genotype

Restriction analysis by Hinf I was achieved after isolation of genomic DNA and PCR optimization. Eighty samples were randomly genotyped using Hinf I enzyme digestion and used as positive controls for HRM. Figure 1 shows the gel image of Hinf I enzyme digestion.

### Genotyping by HRM

All samples were genotyped using HRM analysis. This study was the first and primary study in Pakistan and provides a basic guide of initializing septal defects patients genotyping using HRM. Internationally, this method is widely used to screen genotyping for diagnostic purposes. For genotyping, raw data of amplification plots and melt peaks/curves were sorted, then data points were analyzed via Precision Melt Analysis™ software. HRM genotypes were confirmed by Sanger sequenced RFLP genotypes and were used as positive controls.

### Genotypic frequencies

Genotypic frequencies of cases with controls were compared, which showing that cases (26%) with heterozygous mutant (CT) genotypes were higher than the controls (14.3%). It was mentioned earlier that a loss of enzyme activity around 30% in heterozygous mutant state [17, 18] causes a significantly reduction in homocysteine levels [19]. Moreover, wildtype had high frequencies in control samples, showing its highly resistance toward the congenital septal defects. Besides this, on comparison between 1000 genome project phase III estimated frequencies of this mutation and data from this study data which was 0.26 (Table 4), it was observed that there are similarities in both values; therefore, it was assuming that there is loss of enzymatic activity and may these individuals be survive from some kind of diseases (associated diseases are mentioned in Table 4).

**Table 4:** Statistical analysis of the study

CHD	Genotypic frequencies		Allelic frequencies		*p*-value
	CC	CT	C	T	
Controls	0.85	0.14	0.9285	0.0714	> 0.05
Congenital septal defect	0.74	0.26	0.8698	0.1301	
*Phenotypic relation*					
ASD	0.6956	0.3043	0.8478	0.1521	
VSD	0.7272	0.2727	0.8636	0.1363	
AVSD	0.8235	0.1764	0.9117	0.088	

Furthermore, during this study, mutant (TT) genotype was not found, natural selection might be the reason, which occurred in case of mutant (TT) genotype. Additionally, 1000 genome project phase III reported that no TT genotype was found in their genomic study. Few Asian studies had summarized that homozygous mutant (TT) genotype and the T allele were significantly associated with recurrent pregnancy loss [20, 39, 40] and preterm birth [41], and defending the assumption of the current study. Studies summarizing these topics have not been reported for the Pakistani population.

### Allelic frequencies

**Fig. 2:**
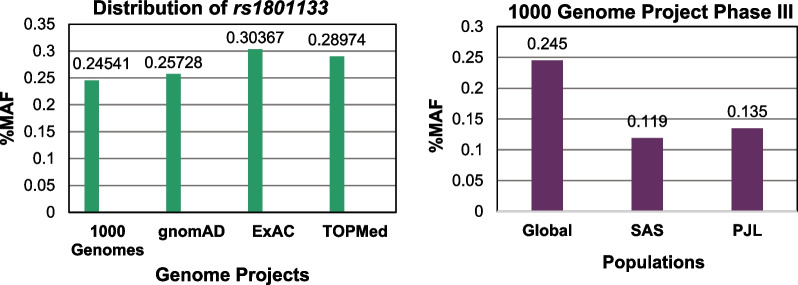
Allelic distributions **a** Contrast of minor allele frequencies (MAF) between genome projects. **b** Division of MAF values among; global = population of world, SAS = South Asians and PJL = Punjabi in Lahore, Pakistan. (*Source*: Ensemble Genome Browser)

Allelic frequencies of cases and controls were compared, which showing an effect of mutant (T) allele to the disease. It was found that the T allele was prone to septal defects individuals (13%) than the control group (7.1%). Further, the allele frequency of Pakistani population estimated by the 1000 genome project was used as reference, finding that it was similar to allelic frequency of mutant allele of patients (Fig. 2b). Moreover, wildtype C allele had high frequencies in both groups, but it was more common in control group, showing the influence of T allele and its contribution to the pathogenesis of disease. Furthermore, a study from Pakistan found an association of this T allele with hyperhomocysteinemia [22]. In addition, studies from China and Egypt have been reported significant association of T allele with CHD [23, 24]. Beside this, *MTHFR* gene variation (*rs1801133*) has been found to be associated with different diseases in different populations.

### Phenotypic relation

Phenotypic data showing that heterozygous (CT) genotype has highly prone to ASD (30.4%), VSD (27.3%) and AVSD (17.6%). Moreover, individually, these septal defects groups had higher percentages than the control group (14.3%), showing that the heterozygous mutant (CT) genotype was susceptible to each septal disease. Furthermore, allelic data illustrated that T allele has least chance to cause AVSD (8%) than VSD (13%), followed by ASD (15%) (Table 4). Further, the MAF value of Pakistani population and South Asian population was lower than the allelic data of ASD and VSD (Fig. 2b), demonstrating the role of mutant (T) allele in pathogenesis.

### Association analysis

Fisher’s exact test using SPSS v 17.0 was used to perform association analysis. The *p*-value was > 0.05, suggesting that rs1801133 (*C677T*) was not statistically significant association with congenital septal defect.

## Conclusions

This study made following assumptions on the basis of experiments and observations; *MTHFR 677C* > *T (rs1801133)* has not statistically significant association with congenital septal defect patients. The present study has not found any homozygous mutant genotype in the duration, suggesting that it may be the cause of natural selection process in the current study population, *i.e.,* patients enrolled in Karachi tertiary care hospital. High-resolution melting (HRM) genotyping was successfully established for known SNP *rs1801133* (*MTHFR C677T)* with the confirmed sequenced and RFLP results. HRM is simple, easy, cost-effective, required minimum time and low amount of chemicals and equipment with higher sensitivity and specificity. This study is the recent update of mutational analysis in Pakistan with complete optimization of protocol for SNP detection. Moreover, parental tobacco consumption was concluded as one of the alarming epigenetic factors in pathogenesis of congenital septal defect. Further, Sindhi and Pashtun populations were found to have more frequent cases of septal defects among the other ethnic groups in the current study.

### Supplementary Information

**Additional file 1: Fig. S1.** Sanger sequencing of wildtype homozygous genotype. **a** There was no change of nucleotide observed in wildtype homozygous; therefore, no change was observed in amino acid codon (**b**). Whereas electropherogram analysis **c** showing one peak confirming the sample had wildtype homozygous genotype (CC). **Fig. S2.** Sanger sequencing of heterozygous mutant genotype. **a** Nucleotide substitution observed in heterozygous mutant sample which change the coding sequence GCC to GTC causing amino acid alteration from **b** alanine to valine. Whereas electropherogram analysis **c** showing two peaks confirming the sample had heterozygous genotype (CT). No TT genotype observes in random samples.

## Data Availability

All relevant data generated during the present study are included this published article.
