# Supplementary material for: Congenital septal defects in Karachi, Pakistan: an update of mutational screening by high-resolution melting (HRM) analysis of MTHFR C677T
Source: Hum Genomics. 2024 Jan 29;18:6. doi: 10.1186/s40246-023-00566-5 (PMC10826090; doi:10.1186/s40246-023-00566-5)
Supplement: Supplementary file 1 — Additional file 1: Fig. S1. Sanger sequencing of wildtype homozygous genotype. a There was no change of nucleotide observed in wildtype homozygous; therefore, no change was observed in amino acid codon (b). Whereas electropherogram analysis c showing one peak confirming the sample had wildtype homozygous genotype (CC). Fig. S2. Sanger sequencing of heterozygous mutant genotype. a Nucleotide substitution observed in heterozygous mutant sample which change the coding sequence GCC to GTC causing amino acid alteration from b alanine to valine. Whereas electropherogram analysis c showing two peaks confirming the sample had heterozygous genotype (CT). No TT genotype observes in random samples. [file 40246_2023_566_MOESM1_ESM.docx]

**Additional File**

Reference Sequence

**(b)**


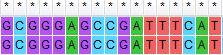

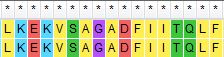

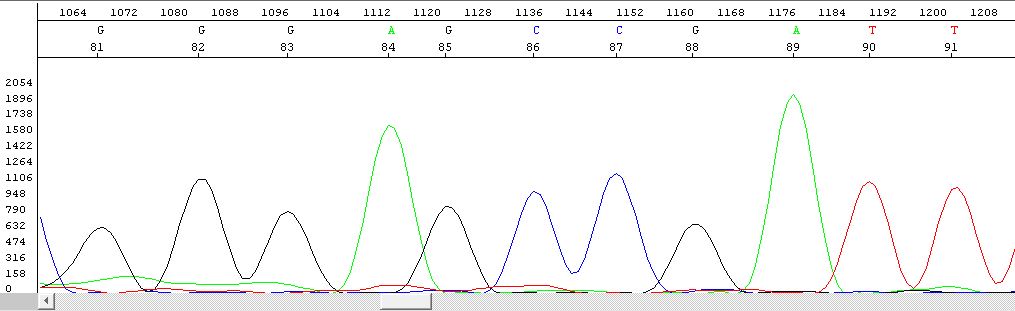


C

***No Change***

**(a)**

**Wildtype Homozygous CC**

Mutant Allele Sequence

Reference Translated Sequence

Mutant Allele Translated Sequence

**Figure S-1: Sanger sequencing of Wildtype homozygous genotype.** (a) There was no change of nucleotide observed in wildtype homozygous, therefore no change was observed in amino acid codon (b). Whereas electropherogram analysis (c) showing one peak confirming the sample had wildtype homozygous genotype (CC).

**(c)**

Reference Sequence

Mutant Allele Sequence

***677C>T***

**(a)**


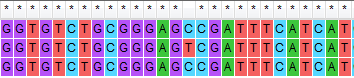

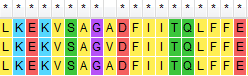

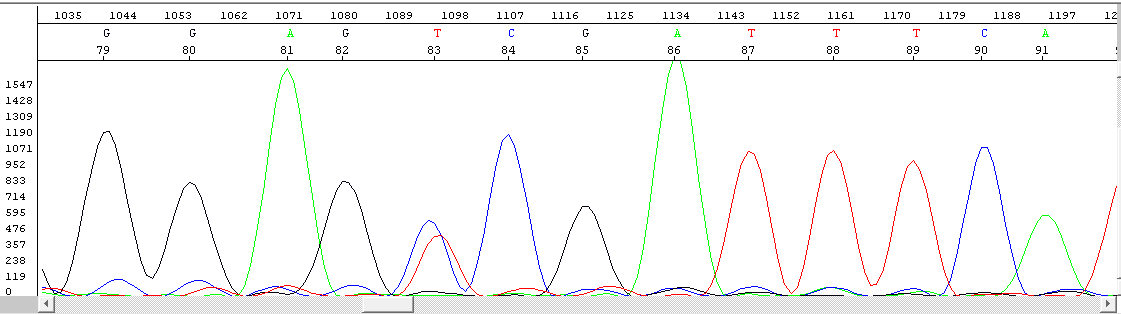


C

T

**(b)**

***226Ala > Val***

Reference Translated Sequence

Mutant Allele Translated Sequence

**Figure S-2: Sanger sequencing of heterozygous mutant genotype**. (a) Nucleotide substitution observed in heterozygous mutant sample which change the coding sequence GCC to GTC causing amino acid alteration from (b) alanine to valine. Whereas electropherogram analysis (c) showing two peaks confirming the sample had heterozygous genotype (CT). No TT genotype observes in random samples.
